# The Internet as a New Tool in the Rehabilitation Process of Patients—Education in Focus

**DOI:** 10.3390/ijerph120302373

**Published:** 2015-02-23

**Authors:** Erzsébet Forczek, Péter Makra, Cecilia Sik Lanyi, Ferenc Bari

**Affiliations:** 1 Department of Medical Physics and Informatics, University of Szeged, Faculty of Medicine, Faculty of Science and Informatics, Korányi fasor 9, Szeged 6700, Hungary; E-Mails: forczek.erzsebet@gmail.com (E.F.); makra.peter@med.u-szeged.hu (P.M.); bari.ferenc@med.u-szeged.hu (F.B.); 2 Department of Electrical Engineering and Information Systems, University of Pannonia, Egyetem Str. 10, Veszprem 8200, Hungary

**Keywords:** information management, information organisation gap, education of informatics, healthcare information, Internet, medical databases

## Abstract

In the article we deal with the rehabilitation of patients using information technology, especially Internet support. We concentrate on two main areas in the IT support of rehabilitation: one of them is the support for individual therapy, the other one is providing patients with information, which is the basic step in emphasising individual responsibility. In the development of rehabilitation programmes, the knowledge of the IT professional and the therapist, in the IT support of web guidance, medical expertise plays the primary role. The degree of assistance involved in the rehabilitation process depends on the IT knowledge of medical (general practitioner, nursing staff) professionals as well. The necessary knowledge required in healing and development processes is imparted to professionals by a special (full-time) university training. It was a huge challenge for us to teach web-based information organisation skills to doctors and nurses, and it is also a complex task to put forward such an IT viewpoint to information specialists in order to create the foundations of the cooperation between IT and healthcare professionals.

## 1. Introduction

In public health, probably the greatest potential provided by the Internet lies in the global availability of information [1]. The sudden development of transfer and storage media made it possible to establish central information, communication and newscaster centres that collect local information and make it available worldwide in a format that is made independent of time, place and the reporting staff [2]. These global centres create the opportunity for validated, reliable healthcare information supply [3].

### 1.1. The Emergence of the Internet in Health Care

With the appearance of health community networks, forums, blogs and other community spaces have come to the foreground. Their significance lies in the community-forming character of individual initiative and in their enormous influence on public opinion. Forums or blogs may represent the notes of a healthcare institution, a group of doctors or an individual, and may focus on general health or on personal problems. The decentralisation of health-related information also poses dangers as professional control of the information is weaker and patients may misuse the information they find [4].

The spread of the Internet not only provides new opportunities for disseminating information and for professional developments, but it also gives rise to new forms of healthcare and makes doctor-doctor, doctor-patient, patient-patient relationships independent of geographical location, thus laying the foundations of telemedicine [5,6]. To provide a wide range of validated professional healthcare information that can be used in personalised primary, secondary or tertiary prevention, in healing itself and in rehabilitation might be considered an aspect of telemedicine [7,8]. Although telemedicine has enormous potential, in Hungary several factors keep this potential from being unlocked and personalised information from being turned into daily routine: amongst others, the lack of support systems, the low level of IT skills of healthcare staff and the lack of medical awareness of IT professionals. (by support systems we mean the medical expert, decision support or information systems that help the physician obtain group or personalised information from professional knowledge bases).

The disappearance of geographical borders, the device independence and the interactivity of the Internet may persuade healthcare staff to take a new role in the future, and they may open up new perspectives in the areas of cure, research and education [9]. Amongst others, the connection (support systems) of these three (centralised, decentralised and personalised) medical information and the demand for their professional supervision emphasises the necessity of further professional development of healthcare staff and medical students as well as of the education informatics specialists in the field of medical information organisation, taking into consideration that exploiting the potential of the Internet in public health is by no means automatic: it needs sober-minded decisions and solid knowledge [5]. (Henceforth by information we mean any form of information disclosure, such as communication, newscast or the description of the therapy compiled by the doctor.).

The development of the “surface web” has rearranged the circle of agents and two poles have taken shape. From an informatics point of view, the difficulty of web development lies in the constantly changing nature of the web, the continuous variation of development tools and minimal development time and the need to be familiar with web applications, which spotlights the informatics expertise and the system developer information specialist. This serves as one of the poles. As a result of place and time independence of information the acquisition and dissemination of medical knowledge became open and global. It was a big step forwards that professional agents have been allowed to take a bigger part in Internet developments. Today, health-related content on the “surface web” is primarily contributed by those of the medical profession. Medical specialists represent the other pole.

The gap between the information specialist responsible for the development of the content and the medical specialist providing the content is gradually being overcome with more intelligent and user-friendly software systems. The implementation details are largely concealed from the clients by development tools through the application of platform-independent standards (such as the recommendations of W3C), so simpler web content can be produced without high-level programming skills. The two key factors, the priority of content and the ease of use of the development tools underlines the importance of the medical professional in the process of providing the content. The tools themselves, however, cannot provide the fundamental system design and organisation considerations and the definition and analysis of the goal, target group and implementation devices of the system.

The wide availability and integration of web development tools are pushing the IT specialist farther into the background (but their role does not decrease) to areas where device integration does not provide health care professionals with sufficient results. One example is system development to support medical rehabilitation, where the direct role of the IT specialist increases. The use of measurement tools and the transformation of virtual worlds into media of therapy require both significant IT skills and medical expertise. This brings the dialogue between the two professions to the fore.

### 1.2. The Information Background of Individual Therapy and Orientation

The rehabilitation of patients in the acute hospital phase takes place with the direct personal control of medical staff, and usually continues at the patients’ home. The definition of individual therapy and intervention is the medical specialist’s task, but in the implementation other professionals are involved. In home care, the family, the general practitioner and the nurses occupy a prominent role, but alongside them, the help and active work of the professionals of the education and the social sector is also required. Besides professionals of the different areas, information technology devices, such as telemedicine and at the same time the Internet play an increasingly important role [10].

Telemedicine devices are used more and more frequently in rehabilitation. Apart from monitoring and measurement devices, individualised therapies using virtual reality technologies are becoming increasingly popular, since they serve as excellent tools in psychiatric, cognitive and physical therapies, and can also be used effectively in gerontology rehabilitation, which has become prominent with the progressive aging of the population [11].

Beyond therapeutic treatment, informing the family and the patient of the disease, the risk factors and the possible prevention can assist the healing process, give a greater chance of avoiding the re-emergence of the disease and can also help the family and the patient adapt a healthier lifestyle. The most obvious tool for the dissemination of information is the Internet. The web provides an excellent organisation, supervision and communication interface, and can thus alleviate somewhat the patients’ complete isolation from the outside world.

However, we are confronted with numerous problems in the practical use of IT tools. One of the problems is the incomplete web knowledge of professionals involved in or managing rehabilitation. The other main problem, the want of deliberate planning in the development of IT tools, can also stem from the lack of knowledge. As a consequence of these, we can observe a widening semantic gap.

## 2. Research Framework

In medical practice, the contribution of doctors makes a huge amount of information available on the Internet today, yet sometimes the retrieval of even the plainest of data can prove a difficult or sometimes impossible task. We can hardly find a program or database that would support rehabilitation, as those which do exist are mostly parts of the ‘deep web’, and thus remain hidden from search engines. The rehabilitation programs and internet sites that can be found and are in use often take neither the guidelines of medical profession nor the specific needs of the target groups into consideration. Consequently, patients practically cannot access the functions of programs or make sense of the information found. We feel that the massive increase in the amount of information available on the Internet does not bring a proportional increase in the opportunities of acquiring useful information.

### 2.1. The Role of Developers’ Knowledge

The supervision of medical information is ensured by the presence of medical professionals in different channels of the Internet. Only healthcare workers, primarily doctors, can refute fallacies or misinformation fuelled by economic interests and provide relevant information. At the same time, medical professionals develop primarily free text applications using development systems, which, although facilitate development, cannot substitute for the proper information technology considerations relevant to supposed target groups and to the specialities of the given medical topics. IT professionals are not familiar with the characteristics of the patients and the diseases, so the systems that they develop do not always meet the actual needs of the patients. Because of this, it is possible that despite being professional in implementation and providing an exact professional description, the actual vehicle and method of presentation, lacking attractive multimedia elements, will be alien to the target group. The fact that neither the textual representation currently dominant in the surface web nor the development tools force the developer to tailor the presentation methods to the target group also exacerbates this problem. This way, even the more intelligent and user-friendly development environments and the work of erudite IT specialists will not bring us closer to our goals because of the lack of knowledge in presenting information in a structured manner in application development.

We can safely conclude that harmonising the presentation method with the presumed target group requires deeper knowledge of several healthcare areas of the IT professional and better information organisation skills on the part of the doctor and the nurse. These considerations make the need for training in medical information organisation unquestionable for both groups.

### 2.2. Components of Information Organisation

We also investigate the consequences of the inadequacy of information organisation in another project aimed at Hungarian stroke websites. In this project, we have analysed the information content, structure and quality of more than 200 websites, assessed the information dissemination efficiency of these web pages with the help of 300 students, and interviewed doctors (*n* = 120) and medical students (*n* = 150) about the best web pages. Our findings so far underpin our previous hypotheses that as a result of popularisation, the presentation of information has become more colourful, but formalisation steps needed for machine processing have lagged behind; instead, free-text and multimedia presentation spread. As a result, breaking the former dominance of structured (database-driven) systems, unstructured or semi-structured systems have emerged, wherein the storage structure does not reflect the structure and properties of the information or wherein the latter are obfuscated by the closed structure of the storage format (e.g., jpg files). Due to the lack of formalisation in the retrieval of information, our presence, our information processing and organisation abilities are also required—which further underlines the vital importance of professional knowledge [12].

**Figure 1:**
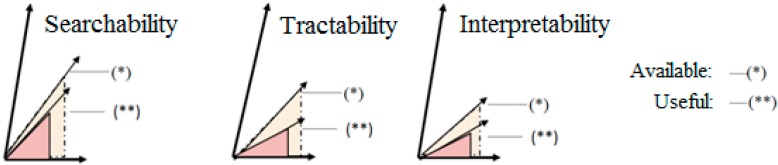
The three main components of information organisation.

In Figure 1, we divided the gap caused by fundamental deficiencies of information organisation into three parts. The first is searchability, the quality of which depends on the web knowledge of the user and the metadata available for the search engine. The two together influence the amount of the useful data, that is, the amount of the available data. Despite all efforts, recently “*ad hoc*” text developments and undocumented multimedia elements have come into foreground. These do not fulfil neither the formalisation requirements necessary for machine “understanding” nor elementary system design or information organisation requirements. Unfortunately, these constitute the main body of the “surface web” visited by search engines, thus the lack of proper information organisation expertise further widens the information organisation gap between the developer and the machine, and, as a consequence, the semantic gap between the user and the machine. This gap is not significantly reduced by the “folksonomia”, or public labelling preferred by Web 2 [13]. All this applies to the “surface web”, but the situation is not better regarding programs and databases applicable in rehabilitation either. Databases in the “deep web” are not included in the scope of search engines, and the ones that are accessible to search engines do not have a page rank high enough to show up in searches.

The popularisation mentioned above also affects the physical tractability of websites already found and the interpretability of the information that can be found there. The interpretability of a webpage depends on the prior knowledge of the target group and method of presenting the information. Tractability affects most of the target groups, as these are medical websites we consider. The tractability of websites is closely linked with the accessibility and the standardisation managed by W3C.

For all three components, the training of developers and users within the framework of medical informatics education can increase the amount of useful information available and the efficiency of online information supply.

## 3. Educational Framework

Medical informatics was born when two knowledge-intensive scientific areas met. Two important criteria of being knowledge-intensive are the creation and the use of knowledge, which have to be integrated not only into science but also into everyday practice. Today we often employ knowledge-intensive devices, which, although they rely on the newest research and development results, generally keep these hidden from users, thus their use requires only little added knowledge (we call a scientific area or device knowledge-intensive if it contains significantly more knowledge than average and uses this knowledge for the enlargement of its own knowledge). Certain knowledge-sensitive areas, such as the use of the Internet, are exceptions. Only people in possession of both the theoretical and the practical background of the Internet as an IT tool can take maximum advantage of its potential.

This knowledge-sensitivity is valid for establishing knowledge and the extraction and procession of the information thus created, which means that the knowledge of developers and users is decisive. Beyond knowledge sensitivity, constant change and the accumulation of knowledge are decisive parts of the given scientific areas and thus of education as well. Since the Internet contributes to the intensive development of healthcare, especially that of telemedicine, it is essential that medical professionals should also be able to play an active role in the developments in addition to using the Internet to its full potential.

### 3.1. Recommendations of Health Care Organisations

With knowledge-intensiveness and constant change in view, informatics teams of medical professional organisations (e.g., IMIA, AMIA, EFMI) make recommendations [14,15] and professional directives. In these recommendations, efficient and uniform education of informatics appears as an ever-pressing need [16,17,18]. We have to take into consideration the development of user-friendly web techniques, the emergence of information and communication centres, the requirements of the decentralised Web 2.0 and especially those of telemedicine, which in intensive development [1]. Although members of professional organisations and people in daily life agree that the efficient use of web requires that medical professionals should play a central role [19], trainings in the use of the web knowledge are sparse.

### 3.2. Two Poles in Hungarian Informatics Education

IT-oriented students learn the design, feasibility criteria and tools of local information systems, the relationship between data, metadata and metadata systems, the structure and functions of machine-close systems and user frameworks.

For students not of the IT profession, such as prospective doctors or nurses, in the case of classical education topics, such as file management and database management, it is enough to know the main functions of the given system. To be successful, the user only has to feed the parameters of the case at hand into the system. The education concerning the web is usually confined to questions of formal searches, the description of the most important databases, the configuration of browser and search services and a mention of Web 2 services [15].

In terms of actual teaching practice, the final result is that IT developers (students closely related to information technology) will be familiar with the depths of software development and will be outstanding IT professionals after graduation. Users (e.g., physicians, nurses and teachers) will become familiar with the file, database or document management frameworks and the use of their main functions through a set of examples, and also get an idea of the basic functions of the web [16,20].

However, in both cases the basic health information organisation skills are missing. As a result, few developers are informed about the specific needs of healthcare, and healthcare students and workers are not aware of any of the basic requirements of information organisation [21,22].

### 3.3. Healthcare-Related Information Organisation in Education

Telemedicine systems often contain very special programs and personalised environment, and are usually in direct contact with the patient and their relatives. The direct contact requires that the informatics system should completely be adjusted to the patient and their health condition, and this is determined by the specific knowledge of the IT specialist and by the intent of the nurse (or the doctor) to create a personalised environment. Although the work of the IT specialist and the nurse seem to be located on two opposing poles of information organisation of health care systems, their work is connected through the organisation of health care information.

The application of the knowledge relevant to the three main web-related information organisation components in Figure 1 on a level corresponding to the actual and future role of the students is essential in both areas [15]. In determining the knowledge belonging to a given level, we tried to apply a systematic approach to compile a complete body of knowledge regardless of which areas of the material are connected to the present studies of the students or to the shaping of their healthcare attitudes or to their prospective roles in healthcare. In each case, we arranged the discussion of the theoretical materials (both in contact classes and in individual assignments) on the basis of a particular area of health care.

On the basis of what has been said, we laid down for our course three main areas of objectives, the properties of which are summed up in Table 1. These objectives together shape the topics of the course.

**Table 1:** Educational levels corresponding to given objectives.

Objective	Goals	Technical Devices
*Assisting current studies*	Finding and processing information. Collecting and processing data. Creating documents. Using Web 2 elements.	IT devices: MS-OFFICE + Google + free programs and applications
*Instilling health care-orientated thinking*	Being aware of and able to use health care databases, information sources and communication interfaces on the internet	Schemas, code systems, thesauri, ontologies. Information source: surface and deep web.
*Assisting patient care*	Creating an internet patient environment depending on the target group, adjusted to a specific patient care task	Code systems. Dublin Core schema MS-OFFICE + Google + free programs and applications

In order to determine these levels we introduced the following notations: {N} refers to nurse students, {IT} refers to IT engineering students. To describe the educational content of the given information organisation topics, we characterised the depth of the material and the level of skills acquired from {1} to {6}: {1}: studying existing websites, {2}: applying templates, {3}: independent exercise in contact classes, {4}: individual assignments, {5}: programming, {6}: system design.

The first topic is the level of metadata that improve searchability and describe content attributes; the second topic is the analysis of the concept of accessibility (as defined by W3C) and the third one is the presentation of a professional portfolio regarding information organisation.

#### 3.3.1. Searchability, Metadata

During the course, we show the deviation of the structure of the web from ideal and the opportunity of correction in content search {N}{1}; {IT}{1}. Content organisation skills and the knowledge of search engines helps students access the surface and the “deep web”. Content attributes that improve searchability can be defined for search engines with the help of so-called metadata standards. These standards are usually created for search engines, but with their help we can use metadata to improve, to some extent, the organisation of the web content ourselves as information developers [23] {N}{1, 2, 3}; {IT}{1, 5, 6} (metadata are data about data, information about information, which is a really general notion; its foundations were laid in healthcare and library science, with the establishment of classification, cataloguing and coding systems, one and a half centuries ago). Our aim is to show students that with the addition of metadata and using efficient information organisation, we can turn a heap of partially structured information that lacks a unified syntactic and conceptual structure into manageable and thus accessible units which are ready for further processing [24] {N}{1, 2, 3}; {IT}{6}. With the addition of our human intelligence and knowledge we can also reach the contents of both the surface and the “deep web”. Thus in addition to metadata (primarily annotation) organisation we acquaint our students with search strategies [25]. With the appropriate search terms and keywords, using a thesaurus, knowing where to find “full text” sources and with sufficient “manual labour” we can already make very effective searches, which, successful as they may be, are not nearly enough [26] {N}{1}; {IT}{1}.

#### 3.3.2. Semantic Web

The demand for efficient information has given birth to the concept of the semantic web (primarily for the purposes of communication between machines), bringing several new ideas and directives in the last decades, from ontology proper to free-text annotations [27]. The best known form of describing content features is the 15 content describing elements of the Dublin Core (DC) [28]. The elements of DC enable the precise description of technical terms and the formalisation of free-text annotations [29,30] {N}{1, 2, 3, 4}; {IT}{1, 5}.

To compile a metadata curriculum, besides relying on prior knowledge of students, we also used medical coding methods as content organising tools [31]. In lectures, we take a look at the specialities and requirements of the formalisation of open web informatics systems. We search for examples of the professional ontologies used in healthcare [32,33] and the thesauri and code-systems which are more frequent than ontologies. Of the thesauri in the field of medicine, Medical Subject Headings (MeSH) has become an international touchstone of information retrieval and processing in medical literature and in the related fields. MeSH is a vocabulary that consists of lower-level, higher-level or coordinate technical terms. Its hierarchical tree structure enables limited or extended searches on different levels. Because of its clear, transparent structure and its role in surveying the literature all medical students are required to know it {N}{1, 2, 3, 4}. The two main types of code systems are classification codes and nomenclatures (e.g., ICD and Systematised Nomenclature of Medicine, SNOMED). In education, we address code systems, their generation rules and meaning as special forms of coding {N}{1, 2, 3, 4}.

#### 3.3.3. Web Accessibility

The second topic is the analysis of the concept of web accessibility according to the W3C (W3C: Web Content Accessibility Guidelines 1.0) [34]. The recommendations of the W3C refer to the device use of people with disabilities and serve as a good guideline for creating a knowledge base. Our statements on the implementation of the websites were worked out for the deaf, for people with hearing defects and for the blind and visually impaired, and we expanded them with general criteria which help all users in orientation and in handling the websites {N}{1}. Improving and expanding accessibility are of primary importance because of the large number of ill or elderly people retrieving medical content. Unfortunately, the designers of the ‘surface web’ are not really aware of the concept of accessibility and hardly employ it as one of their design principles. For this reason, we find it very important to draw the attention of future doctors and informatics professionals to the significance of designing accessible web pages {N}{1}.

The recommendations known as WCAG 2.0 (Web Content Accessibility Guidelines 2.0.) [35] made by the World Wide Web Consortium (W3C) help the creation of web content by laying down directives that guarantee accessibility. If we ignore these principles, the semantic gap between our visitors (elderly people, people with disability, people of low education or with technical disability) and the content on our website increases [36].

The aim of the W3C is to come up with recommendations concerning most technologies related to the web, which recommendations are discussed first by a wide range of professional bodies in industry and science. The recommendations regarding web accessibility were formulated in documents WCAG 1.0 and WCAG 2.0.

The WCAG 2.0 determines how the web content can be made accessible for the underprivileged. Accessibility involves a wide range of people with disabilities, including visual, auditory, physical, speech-related, cognitive, language, learning and neurological disabilities. These guidelines make web content more easy to use for elderly people, whose abilities have changed due to aging, and, of course, improve usability for everyday users as well.

In the lecture, we investigate the implementation of the four principles of accessibility:

*First standard*: The content must be perceivable {N}{1, 2, 3}; {IT}{1, 2, 3, 4, 5, 6}.

*Second standard*: Interface elements must be operable {IT}{1, 2, 3, 4, 5, 6}

*Third standard*: The content and guiding elements must be understandable {N}{1, 2, 3, 4}; {IT}{1, 2, 3, 4}.

*Fourth standard*: The content must be constant (robust) enough to be able to work with current and future technologies {IT}{1, 2, 3, 4, 5, 6}.

The second and the fourth principle are primarily taught to IT specialists, whilst the first and the third principle are connected to the interpretability and the structure of websites, so this knowledge is important for everyone.

#### 3.3.4. Dissemination of Information

Amongst the numerous questions that arise during the development of web applications, the most important one is that of the interests of patients. The successful dissemination of information is closely related to the appearance of the site, the way the information was published and the professional depth. The use of multimedia elements and alternative media not only promotes accessibility, but also draws and keeps attention {N}{1, 2}; {IT}{4, 5, 6}.

The content and structure of a website, the quality and quantity of the written text and the multimedia elements are target group-dependent. The characteristics and needs of the target group are defined via the analysis of a hypothetical, virtual target group. To keep the textual information clear, we organise the sequential content units (topics, themes and sub-themes) into a hierarchical structure. With the graphical representation of the content units we get a concept map [37,38] {N}{1, 2, 3, 4}.

We use the concept map as a device for the contextual representation of themes (documents) with the students. We assign navigation to the concept map and we present it through a browser window on the web. The navigation assigned to the given information organisation level and the organisation of the presentation of the structured information are both target group-dependent activities [39,40] {N}{1, 2, 3, 4}.

## 4. Learning Outcomes

In what follows, we introduce the practical outcomes of two courses that we taught at the Department of Medical Physics and Informatics (University of Szeged) and the Department of Electrical Engineering and Information Systems (University of Pannonia). Initially, local ideas dominated the education, and the topics were more optional, but later, as we joined grants such as “Telemedicine-Orientated research in the fields of Mathematics, Informatics and Medical Sciences”, telemedicine came to the fore, and the topics we discuss in these courses are more directly related to nursing. In the last four years we touched upon the main elements of information organisation according to the themes listed above.

### 4.1. Practical Training of Health Care Students

**Figure 2:**
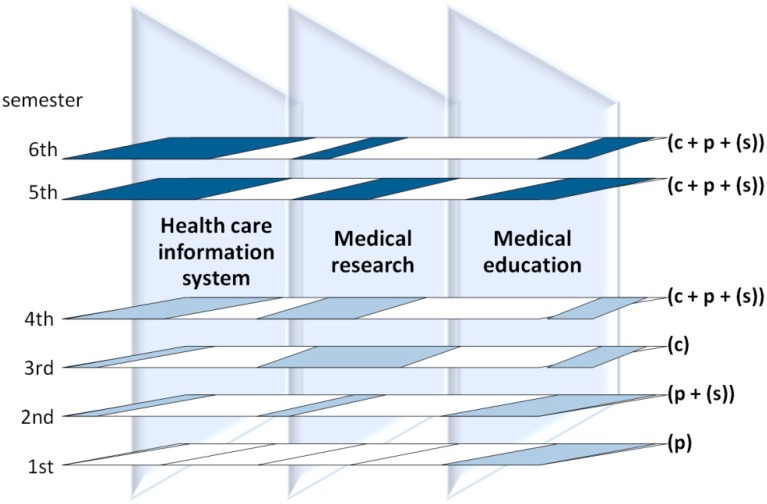
Learning—teaching special model (graduate students of nursing). c: concept, p: practice, s: skills.

In the last four years, we carried out our internet research—more precisely, research connected to the need for web-related education—with the help of 3rd-year graduate students of nursing from year to year (studies in the faculty of health sciences last four years and focus primarily on nursing care), within the framework of the medical informatics course that is scheduled for the 5th and 6th semesters. The entry-level competences of the course are fundamentals of informatics taught in the 1st and 2nd semesters and research methodology taught in the 3rd and 4th semesters. The medical informatics courses encompass both theoretical knowledge and practical skills in three areas of health care, but in different proportion over the semesters. Figure 2 shows the distribution of these components.

In each of the four years, our aim was to create an online professional portfolio [41]. The professional portfolio meant an analysis of a medical topic, which involved the creation of a web questionnaire and the gathering, structuring and a simple statistical evaluation of data. The results gained were supplemented with literary data relevant to the topic, and were processed in the form of a document and a presentation.

This includes the following informatics steps: simple searches centred on a given topic, designing digital and paper-based questionnaires [42], (Google), evaluating and presenting the completed questionnaires, collecting information, editing “large documents” (15–20-page documents, formatted using styles that represent the semantics) (Microsoft Office) and building a portfolio about health care systems and databases. Table 2 shows the schedule in detail.

**Table 2:** The schedule of the 5th semester.

	Contact Hour Focus Topic	Individual Assignment Focus Topic
1–2	Choosing health care “focus topics”, defining the goals and the objectives.	Searching for literature values. Collecting disease prevalence data through domestic patient registry queries
	Surveys on students’ internet searches.	
3–4	The role of search questions and keywords (MeSH) in medical literature databases.	
	Data collection: accessing web content on the surface and the deep web.	
5–7	Using medical code systems (e.g., ICD) and patient registration databases; disease codes, distributions by age and by location.	
	Processing and displaying the collected data.	
8–10	Creating electronic and paper-based surveys, data collection and analysis, comparing the results to regional values.	Creating electronic surveys, data collection and analysis, comparing own results to database values. Preparing a presentation of the results.
11–12	Preparing a presentation of the results in the focus topic.	
13–14	Report on the material learnt in class.	

The exercise is divided into 1 contact hour and 1-h individual assignments per week in two semesters. In the autumn semester, we selected a healthcare topic for a lecture and at the same time for an individual assignment (alcoholism, drugs, stroke, diabetes, HPV, *etc*.), which was elaborated on during the year. In the first (autumn) semester, web information search strategies related to clinical data were introduced and an Internet questionnaire was constructed. In the first and second semester, both code systems and the MeSH thesaurus were used. In the second (spring) semester, in addition to the documents detailed above, case studies of virtual patients were created with the help of online search. During the year, documents created in the lecture and as individual assignments were published in a unified interface [43]. The structured web pages were provided with Dublin Core metadata and then published on an internal website.

At the end of each semester, the semester work was concluded with a report, which primarily meant the completion of a practical assignment. The following results concern only web-based knowledge and are related to two topics, the purposeful search for and publishing of information [44].

#### 4.1.1. Results in Search Strategies

At the beginning of the autumn semester, we surveyed the internet search habits of the students. The parameters which were monitored were the number of search engines that they used, whether they use medical databases and compound keywords. The students observed used only the Google search engine, their keywords were usually simple and they used the first page of the results listed (in this respect, the results did not differ from the results of other surveys and the results of stroke experiments) [45,46]. The same students at the end of the semester still relied mainly on the search engines, but also visited databases (PubMed, The Cochrane Library, DrDiag) and thematic lists (Yahoo Health), thus they accessed the ‘deep web’ information as well. The improvement is obvious, but to assess the improvement rate we must take into account that the initial survey had no stake, whilst the second one was the final test, which entails stronger motivation and more careful work. Yet the change in the direction of the “deep web” is very significant: it means that students begin to move in a wider circle and they also use medical databases [47]. The table shows that in both cases the students got acquainted with the use of databases and thematic lists only on the basis of the information heard at the lectures [48]. The use of the “deep web” is very important also because most of the relevant health information is collected in medical databases, thus being familiar with these databases is essential.

#### 4.1.2. Results in Portfolio Construction and Metadata Organization

In the second semester (for groups 1–4, II.), besides normal information processing, web publishing was also a task in the contact hours, in the individual work and in the final paper. This area, apart from constructing some personal websites, was unknown to the students at the beginning of the semester.

The task of the students was to integrate the text summary, presentation and the questionnaire of the medical topics they elaborated on with the online case studies of virtual patients and the evaluation of these, and present them in an online professional portfolio. Table 3 shows the schedule of the 6th semester in detail.

**Table 3:** The schedule of the 6th semester.

	Contact Hour Focus Topic	Individual Assignment Focus Topic
1–2	Studying the outward appearance of health care homepages, assessing the professional depth of the information, the dissemination method and the accessibility of the content (multimedia elements and alternative media).	Establishing internet appearance: applying a portfolio schema. Uploading images, videos and descriptive text. Preparing case studies. Creating a large document (web literature, statistics, diagrams), applying the required style sheet. Publishing the case studies, the presentation and the large document: information organisation, content organisation (**DC form fill-in, DC elements code insertion).**
3–4	Web: internal structure and external links, the significance of the PageRank value. The relationship between search terms and keywords, the role of metadata. The Simple Dublin Core (DC). Organising the content units (themes, topics, subtopics) into a hierarchical structure and representing them graphically. Establishing the internet appearance: applying a portfolio schema.	
5–6	The potential and the dangers of using homepage sin nursing. Preparing nursing case studies with the help of internet data. Publishing case studies: information organisation, content organisation (**DC form fill-in, DC elements code insertion).** Publishing the presentation: information organisation, content organisation (**DC form fill-in, DC elements code insertion).**	
7–10	Editing a large document that provides the content background, implementing the required style sheet. Publishing the document: information organisation, content organisation (**DC form fill-in, DC elements code insertion).**	
11–12	Harmonising the elements of the portfolio, revising the internet interface. Strengths and weaknesses of our homepage.	
13–14	Report	

Supplementary task: create a case study of the care associated with a given disease (e.g., alcoholism) if another diseases are present.

The process of the survey: students were given browser window schemas to speed up the completion of the task. The schemas had to be filled in with the current data and the current window properties had to be determined. To illustrate online searchability, the 15 elements of the “Simple Dublin Core” related to the topic had to identified, then these codes had to be inserted in the source code of the schema of the topic [49]. To help them fill in the DC, we can use a template found on the website of the National Library (OSZK), where the source code of metadata is given as well (the numerical values given in Table 4 do not refer to the overall score of the paper, but to the tasks indicated).

The table shows that more and more people can fulfil the task of creating a thematic portfolio year by year. The reason for this probably lies in the stronger emphasis on web patterns in the classes than in previous years. Filling in the Dublin Core schema did not pose any particular problem to the students, but implementing it in source code was difficult for them. The latter was not a compulsory task.

**Table 4:** Hierarchical window-management and publication.

	As Hierarchical Website	DC Form Fill-In	DC Elements Code Insertion	Students in Group
Group 1 (II.)	15	17	2	20
Group 2 (II.)	31	35	6	38
Group 3 (II.)	14	15	2	16
Group 4 (II.)	22	23	4	25

The atmosphere during practical training was good. We had discussions with the students in each class and evaluated the strengths and weaknesses of the work in class. Students considered the brisk pace of the course a weakness on several occasions. At the end of the course, however, when they evaluated the work accomplished instead of the momentary exhaustion, they regarded it as one of the strengths. Year after year students found the course not only necessary and efficient but also interesting.

### 4.2. Education of IT Students Related to Healthcare Information Organisation

For engineering information technology students, the *User interface design* course is an optional subject. The aim of the course is to acquaint the future developers with the proper engineering and technological skills with the capabilities of users with special needs (blind, partially sighted, deaf, physically disabled, mentally handicapped, elderly users). In addition to these, a variety of Assistive Technology devices and opportunities, “Design for All” principles are discussed, to teach students how to develop accessible web applications as future IT specialists. During the semester work, they will get acquainted with and use the WCAG 2.0 standard, which is amongst the course requirements. The one-semester course naturally does not provide all the knowledge required for the design of rehabilitation programs and their environments, but it at least draws attention to the most important design and implementation considerations, and we hope that it will “whet the appetite” of students, influencing them in the selection of their future working areas.

The task of information engineering students for six months is the engineering design and then, as a continuation of this, the completion of their thesis. In the last five years, the authors supervised 36 theses related to healthcare or rehabilitation. The topics cover a rather broad spectrum, from virtual reality-based games for visually impaired children to the treatment of a variety of phobias. These are related to a number of international projects (Game On Extra Time Project [50], StrokeBackProject, [51]). In addition to developing a rehabilitation software, these theses were also comprised of preparing an English user manual. Moreover, the students had to learn how to communicate with future users, therapists, doctors and special education teachers in order to be able to tailor the software to their needs. This way, these students, having completed their formal studies, may become useful members of a project team who have no problem working with colleagues from other professions and of different training.

During the courses students are familiarised with the needs and problems of users with special needs, drawing special attention to the problems of the ageing society, which are also reflected in the use of IT devices, and which represent a social phenomenon in developed countries. Consequently, all the systems they design must offer a solution to this problem. Almost all members of today’s middle-aged generation uses the Internet, and it is likely that they will continue to use it till the end of their lives. But will they be able to do so if these will not be designed with their abilities and needs in mind? Therefore, we find that it is really important to familiarise students with these problems, to make them see with the eyes of the user and learn to communicate with doctors, therapists and nursing staff, in order to be able to participate in joint projects as an engineer. That is why IT students need to learn not only the technical knowledge [52].

## 5. Conclusions

In rehabilitation, like in other IT developments, the crucial question is whether the finished product can effectively be used by the patients or their relatives. In the effective use of information technology, the guidance provided by doctors and nurses has a key role. The quality of this guidance also depends on the IT knowledge of professionals. The critical point of the developments is that the doctors and therapists involved in the development often do not have the minimum web knowledge required for web applications, and the IT professionals lack the information organisation skills related to the rehabilitation of patients with special diseases. The lack of information organisation skills impedes the formulation of the needs that give direction to system development, and prevents the integration of professional needs.

As we have stated in the article, our goal is to draw attention to the significance of internet information organisation training. In the first part of the paper, we pointed out the information organisation deficiencies of medical developments on the basis of the literature and our own research. In the second part, we reported a potential solution, which involved supplementing our existing teaching materials with information organisation considerations.

The education of web healthcare information organisation in higher education facilities of healthcare and amongst IT specialists would enable the communication between representatives of the two areas and improve the IT knowledge of practicing physicians and nurses. In preparing the curriculum, we followed these principles, and we hope that as a result of the training we shall get more efficient medical developments in the fields of healthcare and rehabilitation.
